# Detecting drug diversion in health-system data using machine learning and advanced analytics

**DOI:** 10.1093/ajhp/zxac035

**Published:** 2022-02-08

**Authors:** Tom Knight, Bernie May, Don Tyson, Scott McAuley, Pam Letzkus, Sharon Murphy Enright

**Affiliations:** Invistics Corporation, Peachtree Corners, GA, USA; Invistics Corporation, Peachtree Corners, GA, USA; Piedmont Athens Regional Medical Center, Athens, GA, USA; Piedmont Healthcare, Atlanta, GA, USA; Scripps Health, San Diego, CA, USA; EnvisionChange, LLC, Atlanta, GA, USA

**Keywords:** controlled substance compliance, diversion, drug diversion detection, healthcare-acquired infections, machine learning, patient safety

## Abstract

**Purpose:**

The theft of drugs from healthcare facilities, also known as drug diversion, occurs frequently but is often undetected. This paper describes a research study to develop and test novel drug diversion detection methods. Improved diversion detection and reduction in diversion improves patient safety, limits harm to the person diverting, reduces the public health impact of substance use disorder, and mitigates significant liability risk to pharmacists and their organizations.

**Methods:**

Ten acute care inpatient hospitals across 4 independent health systems extracted 2 datasets from various health information technology systems. Both datasets were consolidated, normalized, classified, and sampled to provide a harmonious dataset for analysis. Supervised machine learning methods were iteratively used on the initial sample dataset to train algorithms to classify medication movement transactions as involving a low or high risk of diversion. Thereafter, the resulting machine learning model classified the risk of diversion in a historical dataset capturing 8 to 24 months of history that included 27.9 million medication movement transactions by 19,037 nursing, 1,047 pharmacy, and 712 anesthesia clinicians and that included 22 known, blinded diversion cases to measure when the model would have detected the diversion compared to when the diversion was actually detected by existing methods.

**Results:**

The machine learning model had 96.3% accuracy, 95.9% specificity, and 96.6% sensitivity in detecting transactions involving a high risk of diversion using the initial sample dataset. In subsequent testing using the much larger historical dataset, the analytics detected known diversion cases (*n* = 22) in blinded data faster than existing detection methods (a mean of 160 days and a median of 74 days faster; range, 7-579 days faster).

**Conclusion:**

The study showed that (1) consolidated datasets and (2) supervised machine learning can detect known diversion cases faster than existing detection methods. Users of the technology also noted improved investigation efficiency.

**Key Points**

- Drug diversion creates risk for patients, clinicians, and health systems and is a tremendous challenge fueled by the opioid epidemic, the escalating cost of medications, and the growing complexity of health-system medication supply chains and clinical workflows.
- Most diversion goes undetected by traditional diversion detection methods, such as monthly usage reports, which suffer from low accuracy, specificity, and sensitivity.
- Machine learning and advanced analytics show tremendous potential for improving early detection and investigative effectiveness and efficiency.

Drug diversion^1^ by healthcare workers (HCWs) is a significant national problem, victimizing patients, HCWs, hospitals, and communities alike,^2-6^ and can occur anywhere in the drug delivery supply chain—from purchasing through patient administration and/or safe disposal.^7^ For patients, diversion can lead to substandard care or infections from an addicted HCW whose job function is impaired by drugs.^8,9^ While significant advances to improve inpatient safety and reduce drug diversion have occurred over the past 40 years through efforts supported by standards and regulatory bodies, including the American Society of Health-System Pharmacists (ASHP),^10^ the Joint Commission, the Drug Enforcement Administration, and state licensure boards of pharmacy,^11^ nursing,^12-16^ and medicine,^17^ any innovation or technology that further reduces drug diversion in hospitals provides substantial benefits.^18^

Precise data on drug diversion in individual hospitals is difficult to obtain.^19^ Due to the clandestine nature of diversion and the culture in place in many healthcare institutions, diversion is often left undiscovered and/or unreported.^20^ Numerous investigations and studies have found that roughly 10% of US HCWs abuse controlled substances, a rate that matches abuse within the US population.^21-27^ The US Substance Abuse and Mental Health Services Administration found most drug diversion by HCWs is not discovered and, when identified, is often not reported or prosecuted.^28^ A study of 140 facilities conducted by Porter Research in 2017 highlighted many of these challenges and indicated that 65% of respondents believe the majority of diversion goes undetected.^29^

Traditionally, hospitals seek to prevent drug diversion by having strict chain-of-custody policies, such as requiring that 2 clinicians sign off on waste destruction. In most hospitals, current methods of detecting drug diversion rely on historical reporting from automated dispensing cabinets (ADCs), such as monthly anomalous usage reports or daily discrepancy reports. These approaches have several significant challenges. They typically look back historically at data over several months, causing them to be slow to detect diversion. They can fail to detect diversion, particularly when the person diverting takes active steps to hide their anomalous usage. They also often erroneously flag clinicians who are not diverting. As a result, there is growing demand to improve drug diversion detection methods in hospitals and other healthcare facilities in order to detect diversion sooner, with fewer false positives and false negatives.^30,31^

This paper describes a novel innovation to overcome these challenges and improve drug diversion detection utilizing (1) data sources consolidated from multiple information technology (IT) sources and (2) machine learning analytics.

## Methods

This research investigated the effectiveness of (1) building consolidated datasets from multiple health IT systems, (2) training machine learning models to detect drug diversion in small datasets, and finally (3) testing if those models could detect known diversion cases faster than existing methods in large historical datasets.

### Study design.

The study, approved by our organizations’ institutional review boards, involved building datasets of clinical data from a total of 10 acute care inpatient hospitals. Hospitals were selected to provide a variety of geographical regions, hospital sizes, IT vendors, and maturity of drug diversion prevention programs. One pediatric hospital was also included. Each hospital participating agreed to provide the necessary datasets to undertake the research study, including their recent known diversion incidents. Table 1 shows the health systems participating in the study and the number of hospitals and inpatient beds for each.

**Table 1.** Participating Health Systems and IT Source Vendors

Health System	No. of Hospitals	No. of Staffed Beds	Vendor for IT Source			
			EMR	ADC and IIS	WHL	ETC
Piedmont Healthcare	1	357	Cerner	Omnicell	McKesson	Kronos
Scripps Health	5	1,404	Epic	Pyxis	AmerisourceBergen	Kronos
Health system 3	1	541	Epic	Pyxis	Cardinal Health	Kronos
Health system 4	3	930	Epic	Pyxis	Not used	Not used
Total	10	3,232				

Abbreviations: ADC, automated dispensing cabinet; EMR, electronic medical record; ETC, employee time clock; IIS, internal inventory system; WHL, wholesaler.

The study was designed to test if the use of artificial intelligence, namely machine learning, could automate currently manual methods for detecting diversion in large datasets and thereby offload time-consuming pharmacy operations tasks, allowing pharmacists to focus on more high-value patient care activities, consistent with ASHP’s statement on the use of artificial intelligence in pharmacy.^32^

### Review of existing methods to detect and investigate diversion.

Interviews with pharmacy and compliance personnel at these hospitals were conducted to document existing methods to detect diversion. All hospitals reported that the work required to detect and investigate diversion largely was the responsibility of the pharmacy department, with occasional support from the compliance department, even though the majority of the diversion cases occurred in other departments, notably nursing and anesthesia.

All hospitals reported similar methods for detecting potential diversion leveraging 2 main techniques: (1) reviewing daily discrepancy reports and monthly anomalous usage reports generated from the ADCs and (2) asking clinicians to report impaired behavior by colleagues. Once suspected diversion was detected, all hospitals reported similar investigation methods: First, the investigator manually reconciled ADC and electronic medical record (EMR) records, which they reported required between 4 and 20 hours, depending on the number of transactions by a particular clinician. If that audit showed further evidence of potential diversion, the investigator proceeded with subsequent investigation steps, such as interviewing the clinican suspected of diversion and/or their supervisor.

These existing methods had detected a total of 22 cases of confirmed diversion, which were studied in more detail as discussed below.

### Data collection and consolidation.

Each participating hospital provided electronic transaction data extracted from the source IT vendor systems shown in Table 1. Electronic transaction data included 5 types of IT systems, varying by hospital: (1) EMR data included medication administration records (MARs) and a variety of other clinical EMR information; (2) ADC data included dispense and waste transactions and a variety of clinical and nonclinical information (both profiled and nonprofiled ADC data were included); (3) internal inventory system (IIS) data included medication movement transactions into and within central pharmacy facilities, such as into a central narcotic vault; (4) wholesaler (WHL) data included purchases of medications from the wholesaler and/or registered 503B compounding facility, and other purchasing information; and (5) employee time clock (ETC) data included clock-in and clock-out transactions for employees and other clinicians.

Data consolidation was essential to allow the analytics to track medication movement transactions from wholesaler purchases through patient administration, along with any waste generated.^33^ Data consolidation also allowed a variety of novel cross-system input features to be tested, which would not have been possible without consolidation. Examples included: (1) reconciling WHL shipments with receipts in the IIS to detect if full or partial shipments had been diverted during shipment from the wholesaler by couriers or during receipt at the hospital by pharmacists; (2) reconciling IIS movement of medications from a central pharmacy to the restocking of an ADC to detect if some or all of the quantity moved had been diverted by pharmacy technicians; (3) reconciling ADC dispenses with EMR documentation of administration and any subsequent ADC wasting to detect if some or all of the quantity dispensed had been diverted by nursing or anesthesia providers; (4) comparing clock-in and clock-out ETC transactions with the times of ADC, IIS, and EMR transactions to detect if clinicians had accessed medications at times they were not working “on the clock”; (5) comparing clinical data in the EMR, such as pain score information, with ADC dispensing patterns to detect unusually high dispensing in relation to episodes of high or low patient pain scores.

Significant effort was required to ingest and consolidate the data from these 5 different IT systems because they are typically independent from each other. For example, consolidation required a medication formulary that mapped medication identification numbers in the EMR to medication identification numbers in the WHL, ADC, and IIS systems. In hospitals without single sign-on (SSO) technology, consolidation also required feature creation to enable mapping of username information across the EMR, ADC, IIS, and ETC systems (eg, married name in the EMR mapped to maiden name in the ADC). After the data was consolidated, additional significant effort was required to translate the raw data into meaningful input features for machine learning. For example, to calculate if any dispensed medications were missing, the analytics had to merge the raw ADC dispensed, wasted, returned, and transferred quantities with the raw EMR administered quantities, which in turn required accurately accounting for medication movements across many diverse clinical workflows, such as dispenses from nonprofiled cabinets in procedural areas, and many different dosage forms, such as oral solids, injections, patient-specific infusions, patient controlled analgesia (PCA), and patches. The analytics also were engineered to handle null data elements (eg, outer joins were engineered to retain ADC dispenses without any corresponding EMR administration).

Data was initially extracted as a set of text-based flat files and then loaded using manual data loading routines into a single normalized, relational database, thereby merging these disparate IT systems into a single normalized, relational database. The data extraction and ingestion were subsequently automated to append recent data on a daily basis using a variety of extraction, transformation, and loading (ETL) and enterprise application integration (EAI) methods.

### Definition of candidate predictors and diversion risk factors.

 This research builds on promising earlier studies from a variety of experts showing that analytical systems can detect drug diversion much faster than humans while simultaneously cutting down on effort and user errors.^34-37^ A review of these earlier studies yielded many candidate predictors of diversion. Table 2 shows a list of model input features along with the investigators’ rationale for including each in the testing.

**Table 2.** Definition of Candidate Predictors of Machine Learning Input Features

Category	Input Feature	Rationale for Inclusion in Testing
Timing	Late wasting	Wasting and administration delays could provide time for substitution or tampering. Late was defined as more than 4 hours after dispense.
	Late administration	
Quantity	No administration	Failure to chart the total amount administered, wasted, and/or returned could be diversion. Wasting the full dose, or simultaneous wasting from multiple doses in bulk could indicate falsification of wasting records.
	Partial administration	
	Partial waste/return	
	Full wasting	
	Bulk wasting	
Practice	Handoffs	Gaps in chain of custody could provide opportunities for substitution or tampering. Administering medications in the wrong order or charting the wrong medication could indicate falsification of administration records.
	Incorrect order	
	Incorrect medication	

### Data analysis and machine learning training.

Supervised machine learning^38^ training models were refined iteratively, over the course of a 12-month period, using various datasets, features, and classification thresholds and algorithms. In every iteration, our supervised machine learning methods used one-hot encoded features and split the dataset: 60% of transactions were used for model training, with the remaining 40% of transactions used to test the accuracy of classification.

Initial testing used small datasets from Piedmont Healthcare and a pediatric hospital at a health system designated as Health System 3. These tests investigated various classification methods to assign a numerical risk score to every medication movement transaction, thereby defining transactions associated with a high risk of diversion. We surveyed experienced drug diversion investigators at those facilities, asking them to rank the candidate predictors in Table 2 based on their expert experience, and then applied those survey results to classify selected medication movement transactions as high risk. While initial tests displayed high accuracy, this was largely due to the inherent risks of confirmation bias and overfitting to survey results.

This initial data analysis allowed these 2 health systems to identify several diversion incidents in the initial sample data, prompting an expansion of the scope of the study to include Scripps Health and a fourth health system.

With this expanded dataset, we iteratively tested the accuracy of a variety of machine learning classification algorithms, such as random forest and logistical regression algorithms. To reduce the risk of confirmation bias and overfitting, we introduced K-fold cross-validation and replaced the initial survey results with a heuristic that classified transactions in the 98th percentile as high-risk transactions. (The 98th percentile was selected as a reasonable initial threshold; future analysis will investigate which percentile will maximize accuracy.) Accuracy was improved by using oversampling of high-risk transactions in the dataset, because most transactions were no-risk and thus oversampling the relatively rare high-risk transactions provided more training data for high-risk transactions. We also investigated model multiclass classification using the logistic regression one-versus-all method, but this did not improve accuracy over the single class classification. As a result of these iterative methods, peak accuracy was reached when the machine learning model’s classification of the high-risk transactions in the initial sample dataset was demonstrated to have 96.3% accuracy, 95.9% specificity, and 96.6% sensitivity, and this model was ready for testing against larger, historical datasets containing known diversion cases.

### Testing the timing of detection in historical data containing known blinded diversion cases.

After training of the machine learning model was completed, each health system compiled a list of known drug diversion incidents that had been detected using existing methods prior to the development of the software. These incidents were not used for training the machine learning model but, rather, were used to test the effectiveness of the model, particularly to test (1) if the machine learning models would detect known diversion incidents in the dataset and (2) when that detection would have occurred had the software been available at the time of the diversion.

We then loaded a large historical dataset with 8 to 24 months of history that included 27.9 million medication movement transactions by 19,037 nursing, 1,047 pharmacy, and 712 anesthesia clinicians, including 22 nurses who were known to have diverted medications, as shown in Table 3. The date ranges were selected to end after the latest date of the latest known case at the involved health system and to begin as early as practical given available historical data. Those 22 clinicians were blinded in the dataset and unknown to the researchers conducting the testing. This dataset was loaded into Flowlytics (Invistics Corporation, Peachtree Corners, GA), commercial software for diversion detection installed at the 4 health systems. This software used the machine learning model developed earlier to classify every medication movement transaction with a risk score, thereby classifying a subset of those transactions as high-risk events, simulating how the medication movement data would have been fed into the software had the software been available each day during the historical time period.

**Table 3.** Summary of Scope and Size of Dataset as of July 2, 2020

Health System Name	Medication Movement Transactions	Nursing Personnel	Pharmacy Personnel	Anesthesia Personnel	Nursing Staff Diversion Incidents
Piedmont Healthcare	8,048,549	2,627	134	98	9
Scripps Health	8,047,530	8,097	359	416	7
Health System 3	7,151,551	7,496	521	152	...^a^
Health System 4	4,608,800	817	33	46	6
Total	27,856,430	19,037	1,047	712	22

^a^Health System 3 contributed to the study by providing data and collaborating on input features but did not provide data on known diversion incidents.

Finally, for each of the 22 known diversion incidents, we recorded when the software first flagged high-risk transactions and compared that date to when the diversion had been detected without the use of the software, as described in the next section.

## Results

### Piedmont Healthcare: predictive model evaluation and validation results.

One participating health system, Piedmont Healthcare, assembled a list of 9 known diversion incidents during a 24-month period before these analytics had been developed. Table 4 shows a list of the 9 clinicians who were known to have diverted medications along with the date Piedmont Healthcare first detected and started to investigate each clinician’s diversion.

**Table 4.** Improvements in Diversion Detection Time With Use of Advanced Analytics to Evaluate Documented Diversion Incidents at Piedmont Healthcare

	Date Investigated (ie, First Detected Using Existing Methods)	Date of Earliest Alert by Advanced Analytics Method	Improvement in Detection Time, days
Diversion incident 1	11/9/2015	1/21/2015 8:19 pm	291
Diversion incident 2	1/26/2015	1/1/2015 12:36 am	25^a^
Diversion incident 3	5/4/2016	3/9/2015 5:02 am	422
Diversion incident 4	8/13/2015	1/9/2015 2:12 am	216^a^
Diversion incident 5	5/28/2017	2/14/2016 8:24 am	469
Diversion incident 6	11/23/2016	10/25/2016 11:32 am	29
Diversion incident 7	6/20/2016	1/17/2015 8:28 pm	519
Diversion incident 8	5/28/2017	4/15/2017 9:27 am	43
Diversion incident 9	12/13/2016	5/13/2015 11:19 pm	579

^a^Earliest data extraction data. Had earlier data been extracted, faster detection might have occurred.

The medication movement transactions in that dataset were then classified using the machine learning model, and the transactions entered by the 9 clinicians were inspected to determine if the model had classified any of those transactions as high-risk alerts and, if so, the earliest date of the high-risk alert(s).

As shown in Table 4, the software detected all 9 known diversion incidents at Piedmont Healthcare, and in every case the software detected the diversion faster than it had been detected using existing methods. In short, had the analytics been available during this 2-year period, Piedmont Healthcare’s 9 known drug diversion incidents would have been detected faster (a mean of 288 days and a median of 291 days faster), with detection time improvements ranging from 25 days for case 2 to 579 days for case 9. (Note that the analytics might have been able to detect case 2 even faster if data prior to January 2015 had been available.)

### Scripps Health: predictive model evaluation and validation results.

Scripps Health assembled a list of 7 known diversion incidents during an 8-month period before these analytics were developed, as listed in Table 5.

**Table 5.** Improvements in Diversion Detection Time With Use of Advanced Analytics to Evaluate Documented Diversion Incidents at Scripps Health

	Date Investigated (ie, First Detected Using Existing Methods)	Date of Earliest Alert by Advanced Analytics Method	Improvement in Detection Time, days
Diversion incident 1	8/2/2018	7/11/2018, 18:23	21
Diversion incident 2	5/31/2018	5/19/2018, 04:48	12^a^
Diversion incident 3	8/24/2018	5/29/2018, 17:14	86
Diversion incident 4	10/15/2018	5/24/2018, 07:09	144
Diversion incident 5	10/30/2018	10/22/2018, 16:38	7
Diversion incident 6	10/15/2018	6/2/2018, 11:17	135
Diversion incident 7	10/15/2018	7/16/2018, 00:13	91

^a^Earliest data extraction data. Had earlier data been extracted, faster detection might have occurred.

The software detected all 7 diversion incidents faster than Scripps Health had first detected the diversion (a mean of 91 days, a median of 86 days, and a range of 7-144 days faster), as shown in Table 5.

### Health System 4: predictive model evaluation and validation results.

Health System 4 assembled a list of 6 known diversion incidents during an 18-month period before these analytics were developed, as listed in Table 6.

**Table 6.** Improvements in Diversion Detection Time With Use of Advanced Analytics to Evaluate Documented Diversion Incidents at Health System 4

	Date Investigated (ie, First Detected Using Existing Methods)	Date of Earliest Alert by Advanced Analytics Method	Improvement in Detection Time, days
Diversion Incident 1	January 2018	November 2017	61
Diversion Incident 2	March 2017	January 2017	59^a^
Diversion Incident 3	June 2017	January 2017	151^a^
Diversion Incident 4	February 2017	January 2017	31^a^
Diversion Incident 5	August 2017	June 2017	61
Diversion Incident 6	May 2017	March 2017	61

^a^Earliest data extraction data. Had earlier data been extracted, faster detection might have occurred.

The software detected all 7 diversion incidents faster than the health system had first detected the diversion (a mean of 71 days and a median of 61 days faster; range, 31-151 days faster), as shown in Table 6.

### Investigation audit results.

 Users of the technology also reported that auditing ADC and EMR records required only 10 to 30 minutes with the consolidated dataset; in comparison, auditing required 4 to 20 hours of manual reconciliation using existing methods.

### Summary of results across all hospitals.

Table 7 summarizes the number of incidents detected in each health system and statistics showing how many days faster diversion was detected with advanced analytics and machine learning relative to when the diversion was actually detected with existing methods.

**Table 7.** Aggregated Data on Improvements in Diversion Detection Time With Use of Advanced Analytics to Evaluate Documented Diversion Incidents

		Improvement in Detection Time, days			
Health System	No. of Incidents Detected	Mean	Median	Minimum	Maximum
Piedmont Healthcare	9	288	291	25	579
Scripps Health	7	71	86	7	144
Health System 4	6	71	61	31	151
Overall	22	160	74	7	579

## Discussion

Here we report for the first time how hospitals have integrated various existing IT data sources and implemented machine learning analytics to effectively detect drug diversion. To our knowledge, this is the first published study in which multiple hospitals consolidated data from various healthcare IT systems, such as ADCs and EMRs, for the purpose of detecting drug diversion, and we provide here the first evidence-based examples showing how machine learning algorithms have successfully detected known diversion incidents in blinded data.

These experimental results showed significant improvements in the effectiveness of drug diversion detection as compared to existing methods. This technology detected all of the 22 known diversion incidents from 4 separate and diverse health systems, which included 10 hospitals, over 20,000 clinicians, and over 25 million medication movement transactions. Moreover, the method detected the diversion incidents much faster than they had been detected using existing methods.

Given these results, all participating hospitals expanded their use of the technology, and the National Institute on Drug Abuse funded additional research to accelerate commercialization of the software. This software supports best practices for preventing diversion, as documented in the ASHP guidelines for preventing diversion,^39^ particularly the system-level controls for (1) automation and technology, (2) monitoring and surveillance, and (3) investigation and reporting. The software is also consistent with best practices published by other healthcare organizations, such as the Joint Commission^40^ and the Council of State and Territorial Epidemiologists.^41^

Users of this software report improved effectiveness and efficiency.^42-44^ Automated daily feeds are now in place, allowing the original 10 hospitals, and a growing list of additional hospitals, to see alerts of suspected diversion incidents in near real time, enabling them to start investigations much faster compared to their prior use of monthly monitoring reports and to conduct those investigations more efficiently. The software allows each health system to see patterns of high-risk behavior across all care areas over time. Heatmaps and other visualizations illustrate which transactions, and which clinicians, have been flagged by the software as posing a high risk of diversion. Visualizations include the ability to (1) trend behaviors for the nursing unit, (2) trend behavior against other users on the same unit, (3) trend recorded assessments to identify investigation needed versus practice defects, as well as other clinical anomalies, and (4) conduct additional peer-to-peer comparisons.

Users at these hospitals investigate high-risk transactions flagged by the model, assess if an alert was accurate, and enter that assessment into the dataset, thereby providing external validation of the accuracy of the machine learning model as well as providing an expanding dataset for training. This growing dataset also allows us to occasionally revise the input feature set, discarding features that are less relevant and experimenting with new potential features, thereby reducing the risk of model drift/decay.

As a secondary outcome, users of the software also reported an improvement in investigation efficiency when suspected diversion is detected, with auditing time dropping from 4 to 20 hours of manual reconciliation using existing methods to 10 to 30 minutes with the consolidated dataset.

The combined improvement in both effectiveness and efficiency shows great promise that automating currently manual methods for detecting and investigating diversion using machine learning will offload time-consuming pharmacy operations tasks, allowing pharmacists to focus on more high-value patient care activities.

Health systems are using this software as one key component of their overall controlled substance diversion prevention program (CSDPP). Hospitals need investigations conducted by trained drug diversion professionals and a leadership team of departmental heads in order to protect innocent HCWs and to confirm the software’s alerts. It is worth noting that once a suspected diversion has been detected, the value of an interview is key, and the Mayo Clinic estimates that approximately 75% of suspected diversion investigations have been brought to closure by a confession on the part of the drug diverter.^45^ Treatment programs are vital for impaired HCWs who seek out help, and these programs have a high overall recovery rate when grouped with intensive inpatient management and follow-up care.^46^ These components combined create a robust process for diversion deterrence and prevention.

Moreover, health systems are using this software to expand their CSDPP to not only detect diversion of controlled substances but also to expand their surveillance to include medications that are not controlled substances, particularly medications associated with a high risk of diversion due to their high value. With the increasing cost of medications, as well as the growing complexity of health systems and facilities, all participants in this study recognized the expanding need to monitor their supply chains beyond controlled substances to also include expensive drugs and other frequently diverted substances.

There were several limitations to our study. First, we focused on detecting known diversion incidents rather than detecting previously unknown incidents. The latter will be studied in a future phase by asking the same hospitals that participated in this study to investigate and confirm if the analytics successfully detected previously unknown diversion incidents. Second, the number of known diversion incidents in the study was fairly small (*n* = 22), so the results are based on a fairly small population of diversion incidents. Third, we focused on nursing personnel, and future research will expand to pharmacy, anesthesia, and other clinical areas. For example, in pharmacy we will investigate diversion detection in purchasing, stocking, compounding, repackaging, and returns. Fourth, we tested sensitivity, not specificity (ie, we confirmed that the analytics detected diversion that went undetected by existing methods). Because this study focused on experiments to confirm that the software could detect previously known drug diversion incidents, additional research is currently underway to (1) confirm that the analytics can detect previously unknown drug diversion incidents; (2) measure specificity in order to develop analytics that will yield fewer alerts than were observed in this study through use of the 98th percentile, and to confirm those analytics produce fewer false positives than existing methods; and (3) improve accuracy over time by engineering new input features and adding new data to the machine learning training set. This research will also allow us to measure and learn from false negatives (ie, occasions when the analytics fail to detect diversion that would be detected using other methods, such as a clinician coming forward to self-report). That additional research is being conducted in the same hospitals, as well as additional hospitals that have since joined the study, and will be published when completed.

These results provided visibility to the inadequacy of existing detection methods. The key benefits are (1) detecting diversion faster,^47^ preventing HCWs from jeopardizing patient safety^48-50^ or their own health and careers, and (2) reducing the effort required to detect diversion for hospital administrators, nurse managers, and other supervisors.

## Conclusion

The study showed that (1) a consolidated dataset and (2) supervised machine learning can detect known diversion cases faster than existing detection methods. These analytics detected known diversion cases (*n* = 22) in blinded data faster than existing monitoring systems: a mean of 160 days and a median of 74 days faster (range, 7-579 days faster). Moreover, users of the software have noted a dramatic improvement in investigation efficiency when suspected diversion is detected.
